# Colorectal Cancer Screening and Management in Low- and Middle-Income Countries and High-Income Countries: A Narrative Review

**DOI:** 10.7759/cureus.70933

**Published:** 2024-10-06

**Authors:** Barbara A Abreu Lopez, Rafael Pinto-Colmenarez, Fides Myles C Caliwag, Lorraine Ponce-Lujan, Mariela D Fermin, Ana V Granillo Cortés, Anette G Mejía Martínez, Ismael G Zepeda Martinez, Fernanda Gress León

**Affiliations:** 1 General Medicine, Universidad de Carabobo, Valencia, VEN; 2 Medicine, Universidad de Carabobo, Valencia, VEN; 3 General Practice, Ateneo School of Medicine and Public Health, Pasig, PHL; 4 General Practice, Universidad de San Martín de Porres, Lima, PER; 5 General Practice, Instituto Tecnológico de Santo Domingo, Santo Domingo, DOM; 6 Internal Medicine, Universidad Nacional Autónoma de México, Ciudad de México, MEX; 7 Internal Medicine, Saint Luke Escuela de Medicina, Ciudad de México, MEX

**Keywords:** colonoscopy, colorectal cancer (crc), colorectal cancer screening, high income countries, low income countries

## Abstract

Colorectal cancer (CRC) remains a leading global health challenge, being a highly prevalent cancer and a major cause of cancer-related deaths worldwide. The incidence of CRC varies significantly between high-income countries (HICs) and low- and middle-income countries (LMICs), with higher rates of incidence but lower mortality in HICs. Factors such as genetic predisposition, lifestyle, and dietary habits play significant roles in CRC development, with the Western diet and limited access to screening contributing to increased incidence. This review highlights disparities in CRC screening, management, and outcomes between HICs and LMICs, with HICs benefiting from advanced screening methods like colonoscopy and sigmoidoscopy, while LMICs face challenges due to limited healthcare infrastructure and resources. Tailored strategies, including low-cost screening options and community-based initiatives, are critical in LMICs to improve early detection and outcomes. Future directions for improving CRC care globally include telemedicine, artificial intelligence, and mobile health technologies to bridge access gaps, as well as personalized medicine to enhance treatment efficacy. Global collaboration and investment in healthcare infrastructure are necessary to reduce CRC-related mortality, particularly in resource-limited settings.

## Introduction and background

Colorectal cancer (CRC) is a malignant tumor that typically begins as adenomas, which can progress into malignant lesions [1]. This is the third most common cancer in the world, accounting for approximately 10% of all cancer cases and the second leading cause of cancer-related deaths [2]. By 2030, the number of new cases is expected to reach around 170,968, marking a 17.3% increase from the total cases in 2020 [3]. The development of CRC results from changes in the healthy epithelium of the colon, including the development of adenomatous polyps that may proliferate, grow, and accumulate genetic and epigenetic mutations over time [4]. Genetic factors, such as germline mutations in DNA mismatch-repair genes leading to Lynch syndrome, mutations in the tumor suppressor adenomatous polyposis coli (APC) gene causing familial adenomatous polyposis, or alterations affecting the Wnt/β-catenin pathway, have significant effects on the increase of CRC [5-7]. Environmental risk factors and lifestyle aspects also play a significant role in the incidence of CRC.

Convincing evidence from the World Cancer Research Fund International (WCRF) Continuous Update Project (CUP) concluded that higher consumption of red and processed meat, and lower consumption of dietary fibers, increase the risk of CRC [8]. This association can be explained by the formation of N-nitroso compounds when red meat is broken down in the gut, many of which are carcinogenic [9]. The death rate for CRC between 2018 and 2022 was 12.9 per 100,000 men and women [10]. However, the survival rate can be increased by 90% through early detection and treatment, such as surgery or a colonoscopy [1]. Although survival rates for patients over 65 years of age have improved significantly, they have declined in adults younger than 50 years of age, as screening is not encouraged in this population. This requires public awareness about prevention and early diagnosis, especially in high-risk populations [4]. Even though more than two-thirds of all cases of CRC happen in countries with a high or very high human development index (HDI), a significant increase in both incidence and mortality of CRC is now observed in countries in Eastern Europe, Asia, and South America [11]. Health systems play a limited role in influencing health when other factors, such as the conditions in which people are born and grow up, are considered [12]. The differences in health systems and economies between countries may potentially lead to a lack of equality affecting the early diagnosis and prognosis of CRC [13]. This article compares colon cancer screening and management strategies in low- and middle-income countries (LMICs) and high-income countries (HICs), highlighting disparities, challenges, and potential solutions. 

## Review

Epidemiology and global burden 

**Figure 1:**
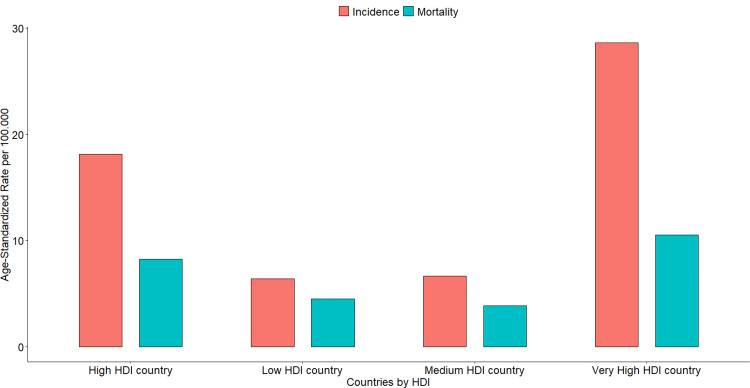
Graphical representation comparing CRC incidence and mortality rates of all countries divided by their HDI. Bar plot presenting the incidence and mortality age-standardized rates of all countries divided by their HDI. Note how countries with high- and very-high HDI have higher incidence and mortality rates, but low- and medium-HDI countries have higher mortality-to-incidence ratios. HDI: Human development index; CRC: Colorectal cancer Source: GLOBOCAN 2022 [14] The figure was created using R Statistical Software (v 4.3.3) [16].

**Figure 2:**
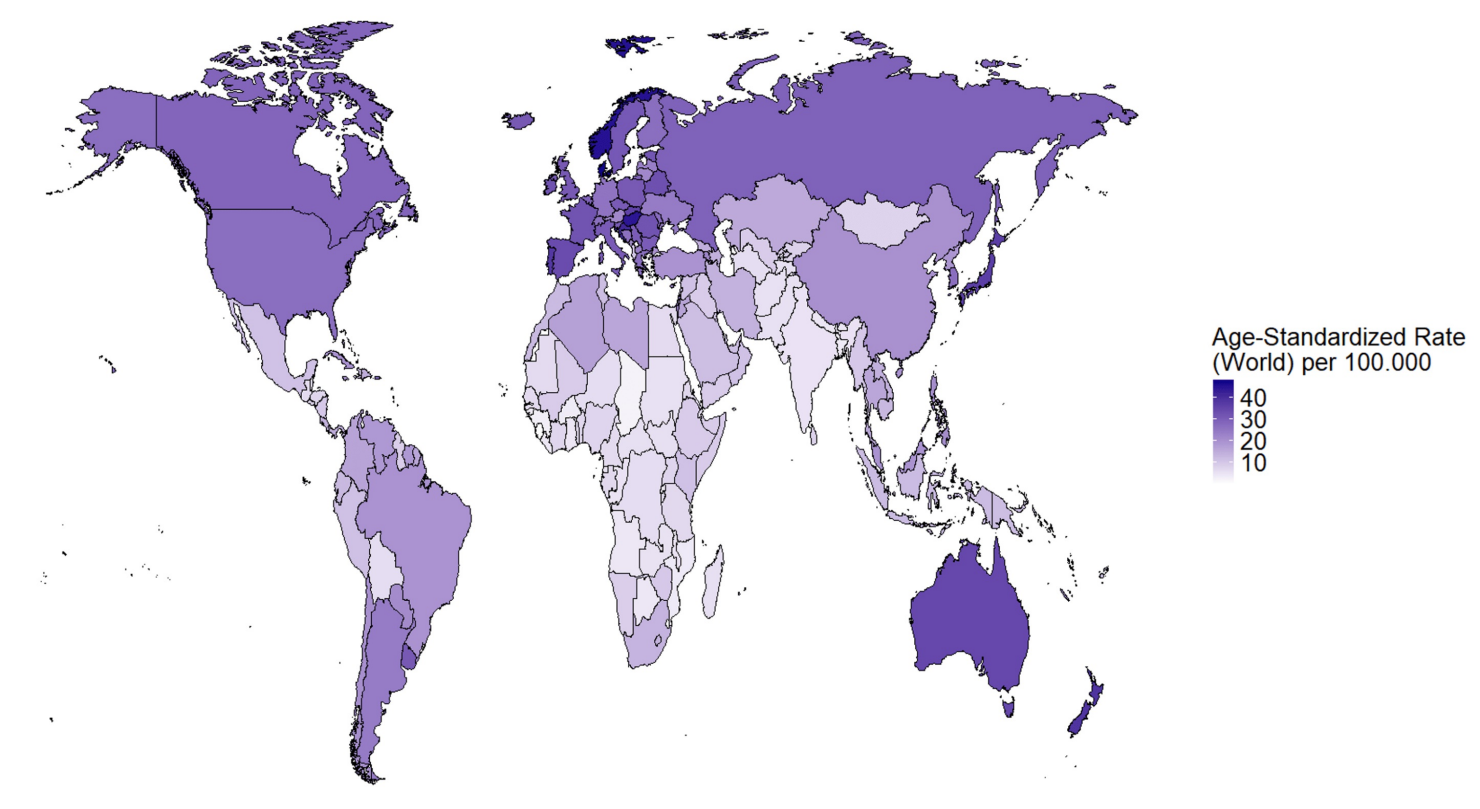
Global incidence of colorectal cancer in 2022, age-standardized rate per 100,000 individuals. A global map presenting the age-standardized rate for the incidence of CRC in each country. Darker colors represent the highest incidence of CRC, while lighter colors represent the lowest incidence. Note how the regions of Australia-New Zealand and Europe have the highest incidence, whereas Western Africa, Middle Africa, and South-Central Asia have the lowest incidence of CRC. CRC: Colorectal cancer Source: GLOBOCAN 2022 [14] The figure was created using R Statistical Software (v 4.3.3) [16].

The incidence of CRC is three to four times higher in HICs compared to LMICs [14]. However, HICs have relatively low mortality rates compared to LMICs, which have higher mortality-to-incidence ratios (Figure 1) [15]. The regions with the highest incidence of CRC are Australia-New Zealand, Northern Europe, and Southern Europe, with rates of 35.3, 32.0, and 31.5 per 100,000 individuals, respectively (Figure 2) [14]. Conversely, Western Africa, Middle Africa, and South-Central Asia have the lowest rates, with 6.5, 5.5, and 5.5 cases per 100,000 individuals, respectively (Figure 2) [14].

Higher exposure to risk factors in HICs [17] and a lack of access to screening in LMICs [18] could explain such noticeable differences across these regions. The WCRF/American Institute for Cancer Research (AICR) reports that alcohol consumption, red and processed meats, increased body fatness, and increased adult attained height increase the risk for CRC. In contrast, physical activity, calcium supplementation, whole grains, foods containing dietary fiber, and dairy products protect against CRC [19]. A Western diet with higher consumption of alcohol and red and processed meats, and less intake of vegetables, fruits, and nuts may also explain the higher incidence of CRC in HICs [17].

However, these trends have been recently shifting. The total number of CRC cases is predicted to reach 3.2 million by 2040 [20], mainly due to increasing incidence and mortality rates in LMICs, especially in Eastern Europe, Latin America, and Asia. In contrast, in HICs, these rates are stabilizing or decreasing [11]. For example, in Australia, the male incidence rate decreased from 48 to 38.9 cases per 100,000 individuals from 1993 to 2017 [14], and the male mortality rate decreased from 20.1 to 9.2 cases per 100,000 individuals from 1993 to 2020 [14]. Similarly, Iceland, New Zealand, and Japan also show a decrease in these rates [11].

HICs have seen a decline in CRC incidence due to population-level shifts towards healthier lifestyles (e.g., physical activity, increased consumption of fiber) and through the introduction of screening and preventive methods, especially colonoscopy and the removal of precursor lesions, significantly reducing CRC incidence since the late 1990s [14]. Conversely, in LMICs, westernizing lifestyle patterns and demographic aging may explain the increasing incidence of CRC [17]. Additionally, limited access to screening can result in a delayed diagnosis, decreasing overall survival; furthermore, a lack of access to treatment options and differences in cultural and health beliefs may also contribute to the increased burden of CRC [20,21].

Screening strategies

CRC screening relies primarily on lower gastrointestinal endoscopy, such as sigmoidoscopy and colonoscopy, for the early detection of potentially cancerous lesions [20]. This approach has demonstrated a significant reduction in mortality rates. Numerous global organizations, including the American College of Gastroenterology (ACG) and European counterparts, endorse regular colon cancer screening [22,23]. The American Cancer Society recommends starting screening at age 45 and implementing tailored approaches for individuals with risk factors [24]. Colonoscopy is the most widely used tool, enabling diagnostic and therapeutic interventions by identifying and removing polyps, which can be further evaluated by biopsy [22,25]. However, the invasive nature of this test is still considered the gold standard screening method. Other noninvasive screening tests, such as fecal occult blood tests (FOBTs), detect hidden blood in the stool, which can be a sign of CRC or large polyps. A fecal immunochemical test (FIT) is based on antibodies that specifically react with human hemoglobin, making it more sensitive and specific than FOBT [26]. There is mixed data about the superiority of each test, and it seems FIT has more acceptance [27]. While these programs have shown success in reducing CRC mortality, coverage rates vary across countries. Factors influencing coverage include program design, access to healthcare, patient awareness, and participation rates [28]. Some countries have achieved high screening rates through effective outreach, patient education, and streamlined referral processes. However, disparities in screening rates often exist based on socioeconomic factors, highlighting the need for targeted interventions to improve equity [29]. 

Overall, population-based CRC screening programs have been instrumental in reducing the burden of this disease in HICs. Continued efforts to increase screening coverage and address disparities are essential to maximize the benefits of these programs [28,29]. Advances in CRC screening offer new options. Capsule endoscopy, a non-invasive method where a tiny camera is swallowed, has shown promise in detecting polyps. Computed tomography (CT) colonography, another non-invasive imaging technique, has demonstrated comparable efficacy to colonoscopy in identifying polyps [30]. These screening methods are more cost-effective in terms of saving lives compared to not conducting any screening. In the case of colonoscopy, the incremental cost-effectiveness ratio (ICER) for colonoscopy was generally below the commonly accepted threshold of $50,000 per quality-adjusted life year (QALY) gained [31]. Several factors contribute to the cost-effectiveness of colonoscopy. The high sensitivity, specificity, and reduced mortality outweigh the costs [32]. However, it also depends on other factors, such as training providers, the cost of the procedure, and other screening options. In regions with low CRC prevalence or limited resources, the cost-effectiveness of colonoscopy may be less favorable. A combination of tests has gained traction in the field with the increasing cost of colonoscopy; for example, annual FOBT with five-yearly sigmoidoscopy [33]. The cost-effectiveness of non-invasive screening tests depends on specific factors, such as prevalence and the cost of healthcare services; stool DNA tests are more cost-effective than other non-invasive tests [34].

Colon cancer is a significant public health burden in LMICs, characterized by late-stage diagnosis and poor prognosis. Implementing effective screening programs is crucial to reducing mortality. However, numerous barriers hinder their implementation [35]. Limited resources, inadequate infrastructure, and cultural and socioeconomic challenges are major obstacles to colon cancer screening in these settings. Lack of trained personnel, insufficient equipment, and poor healthcare access compound the issue. These factors contribute to low screening rates and delayed diagnosis [36]. Given these constraints, the feasibility of population-based screening programs is often limited. Opportunistic screening, where individuals are screened during other healthcare visits, might be a more practical approach and can improve CRC detection rates, albeit with lower sensitivity than population-based programs [37]. Low-cost screening options are essential to address cost-effectiveness and scalability. FITs have gained traction due to their simplicity and affordability [38]. Studies have shown that FITs can be effectively implemented in low-resource settings. Additionally, community-based initiatives involving health educators and community leaders can enhance screening uptake and awareness [39].

Implementing colon cancer screening programs in LMICs requires tailored strategies that address the unique challenges of these settings. A combination of opportunistic screening, low-cost tests like FIT, and community engagement can contribute to early detection and improved outcomes. Further research is needed to optimize screening programs and evaluate their impact on reducing colon cancer mortality. In patients with positive CRC, it is important to screen the liver, as it is the most common site of metastasis. Alterations in the liver can be visualized with imaging studies and laboratory tests, as liver enzymes are not specific. However, when a liver scan is performed without a diagnosis of CRC, it is necessary to determine the source of the abnormalities, as various diseases and conditions can cause them [40-42].

Management approaches 

Treatment in HICs

The treatment of CRC involves a combination of surgery, chemotherapy, radiation therapy, and targeted therapy, depending on the cancer stage and patient-specific factors, according to the National Comprehensive Cancer Network (NCCN) guidelines [43,44]. For Stage I colon cancer, surgical resection is typically sufficient, with adjuvant chemotherapy considered only for high-risk cases. Stage II usually involves surgery, with adjuvant chemotherapy recommended for patients with high-risk features, like T4 tumors or lymphovascular invasion. Stage III treatment includes surgical resection followed by adjuvant chemotherapy, commonly using FOLFOX (folinic acid, fluorouracil, oxaliplatin) or CapeOX (capecitabine, oxaliplatin) regimens, as they effectively reduce the risk of cancer recurrence by targeting cancer cells through a combination of DNA damage and inhibition of DNA synthesis. In Stage IV, treatment is more complex and often includes systemic therapies such as fluoropyrimidines combined with oxaliplatin or irinotecan, along with targeted therapies like bevacizumab, cetuximab, or panitumumab, depending on genetic mutations [43]. For rectal cancer, a multidisciplinary approach often involves neoadjuvant chemoradiotherapy to shrink the tumor before surgery, with postoperative adjuvant therapy based on pathological findings. Regular follow-ups and monitoring are essential for assessing treatment effectiveness and managing side effects [44].

Regarding surgical options, recent advances in minimally invasive surgery (MIS) for CRC treatment in HICs have significantly enhanced surgical outcomes and patient recovery compared to traditional open surgery. Laparoscopic and robotic-assisted techniques represent major innovations in this field, providing several advantages. Unlike open surgery, which requires a large abdominal incision and leads to increased postoperative pain, longer hospital stays, and extended recovery times, MIS uses smaller incisions, resulting in reduced discomfort, shorter hospitalizations, and faster recovery [45]. MIS techniques enhance visualization and precision through high-definition cameras and robotic systems, improving surgical accuracy and reducing complications like blood loss and infections. While both MIS and traditional open surgery aim for similar oncologic outcomes, studies suggest that MIS can achieve comparable or better results with fewer complications and improved patient satisfaction due to less pain, quicker recovery, and better cosmetic results [45].

CRC management in HICs also relies on a multidisciplinary approach to patient care. Oncology teams, composed of surgeons, medical oncologists, radiation oncologists, pathologists, and specialized nurses, work collaboratively to develop and implement individualized treatment plans [46]. Palliative care is integrated into the treatment process from the early stages, focusing on improving the quality of life for patients and managing symptoms such as pain, nausea, and fatigue. In HICs, palliative care teams work closely with oncology teams to provide holistic support, addressing physical symptoms as well as emotional, social, and spiritual needs [47]. Survivorship programs have also gained prominence, reflecting the increasing number of patients living beyond their initial cancer treatment. These programs provide ongoing monitoring for recurrence, management of long-term side effects, and support for psychological and social challenges. Survivorship care plans are often personalized, considering the patient’s treatment history and specific needs, and are coordinated by a multidisciplinary team to ensure continuity of care [48].

CRC treatment in HICs is highly personalized, considering cancer stage and patient factors. Advances in MIS have significantly improved outcomes and recovery compared to traditional open surgery. Oncology teams collaborate to develop tailored treatment plans, integrating palliative care early to enhance quality of life. Survivorship programs address recurrence risk, side effects, and psychosocial challenges. These advancements have led to improved outcomes and enhanced patient well-being, with ongoing research exploring innovative therapies and the long-term benefits of MIS.

Treatment in LMICs

Access to effective CRC treatment in LMICs is challenged by the limited availability of essential treatments and advanced technologies [49]. Inadequate healthcare infrastructure and financial constraints result in a shortage of key resources, like chemotherapy drugs, targeted therapies, and biologics. Many LMICs also lack adequate surgical facilities and radiotherapy equipment, which are usually needed for effective CRC treatment [21,49]. This scarcity results in delayed diagnoses, advanced disease stages at presentation, and poorer patient outcomes. Financial barriers further complicate the situation, as high treatment costs and inconsistent drug supplies prevent patients from maintaining their treatment regimens. The economic burden on patients can lead to incomplete or abandoned treatment. Additionally, a shortage of trained healthcare professionals impairs the delivery of comprehensive care [49].

Resource-stratified treatment guidelines have been developed to address these issues and provide a framework for delivering care in limited-resource settings. These guidelines, such as those published by the American Society of Clinical Oncology (ASCO), offer tiered recommendations based on available resources [50,51], distinguishing between high-resource environments with advanced technologies and therapies and limited-resource settings where cost-effective and basic care strategies are prioritized. In high-resource settings, comprehensive diagnostics include advanced imaging techniques, such as positron emission tomography (PET) scans, and extensive genetic testing. Conversely, basic imaging, like CT scans or ultrasound, and essential diagnostic tests, such as FOBTs, are emphasized in limited-resource settings. Despite ultrasound having lower costs than other imaging tests, it also requires training, even during medical school, which can increase the cost of implementation [52,53].

Surgical approaches often involve laparoscopic or robotic-assisted techniques for precision and minimal invasiveness, making them difficult to implement in low-resource settings. The average hospitalization cost of laparoscopic abdominal surgery in the U.S. is around $16,000 ± $14,800, compared to robotic-assisted operations, which average $18,300 ± $13,900 [54]. In contrast, limited-resource settings rely on traditional open surgeries due to the availability of equipment and limited expertise. HICs can utilize newer regimens and targeted therapies based on molecular profiling for chemotherapy, while LMICs focus on standard, cost-effective drug regimens. Radiotherapy in high-resource settings features advanced methods like intensity-modulated radiation therapy (IMRT), whereas limited-resource environments typically use conventional radiotherapy techniques due to equipment limitations. These recommendations ensure that treatment remains effective and practical according to available resources [50,51].

International collaborations and aid programs have also played an important role in improving treatment access in LMICs. Initiatives like the Global Oncology Leadership Task Force and partnerships between HICs and LMICs have facilitated funding, training, and the provision of essential medical supplies to low-resource settings [55]. ASCO also launched the International Cancer Corps, a volunteer-based program where oncology professionals enhance cancer care in LMICs by integrating palliative care through training and volunteer support, advancing multidisciplinary management with targeted training and tele-education, and improving the quality of care via training on ASCO's quality measurement platform and quality improvement opportunities [56].

CRC treatment in LMICs faces significant challenges due to limited resources, infrastructure, and access to essential treatments. These challenges lead to delayed diagnoses, advanced disease stages, and poorer patient outcomes. To address these limitations, resource-stratified treatment guidelines and international collaborations are crucial for improving access to effective care and enhancing patient outcomes.

Barriers and disparities

Disparities in healthcare infrastructure and resource allocation significantly affect healthcare quality in LMICs and HICs. Several studies have reported limited resources for basic medical attention, such as a scarcity of medical supplies, medications, equipment, and unreliable power, in more than 40% of the LMICs studied, compared to 10% of HICs. Additionally, there was evidence in 54% of LMICs that 38% of their healthcare facilities lacked access to water, 19% had no improved sanitation, and 35% had no soap and water facilities [57].

There was an evident shortage of healthcare providers, including general and specialist practitioners, seen as a factor affecting LMICs in 40% of cases; compared to HICs, this value is reported as half. In both rural areas, there’s a wide disparity in the geographic distribution of healthcare workers and facilities, with long travel distances hindered by poor road infrastructure and/or a lack of transportation [58-60]. Healthcare costs are the main financial burden for the population in both HICs and LMICs. Even though there can be out-of-pocket expenses, such as medication and indirect healthcare costs - including loss of work time, loss of income, and diversion of limited resources - these factors affect LMICs. On the other hand, in HICs, health insurance can be costly in some cases or may lack complete coverage, which would still result in indirect costs [58,60,61].

When analyzing high-income nations, residents in lower-income communities and migrant populations must be considered. Several members of migrant communities don’t access healthcare due to fear of deportation and incarceration. They also face language and communication barriers and have trouble navigating the healthcare system due to a lack of familiarity and the need for additional support [61]. Refugees and asylum seekers may also delay their search for healthcare due to their legal status and the uncertainty of the length of stay in the area. In addition, they may withdraw from the healthcare system due to fear of stigmatization [62]. Several studies have also found that a lack of education and poor health literacy, especially in rural areas, delay the decision to seek care or even reach an adequate facility due to a lack of knowledge and a perceived need for attention, subsequently hindering medication adherence and incurring higher costs due to the misuse of available health resources [58,63,64].

Cultural beliefs and misconceptions about diseases seen as witchcraft or having a spiritual cause rather than a physical one steer some communities in LMICs to seek traditional medicine; therefore, they visit a healer in the community rather than a biomedical provider. This was also seen in migrant populations in HICs [62,65]. Sociocultural perceptions of women from certain communities and religions, along with their lack of decision-making power and expectations of gender roles, reported about LMICs and migrant communities in HICs, make childcare, housework, or caring for a sick relative a higher priority than accessing healthcare services [60,62]. Strategies can be developed to improve healthcare access with a focus on health equity by customizing programs to meet the specific needs of different groups, especially those from lower socioeconomic backgrounds, who may require more services or additional support to achieve the same level of healthcare utilization as others [66]. Subsidized services are offered in rural communities in HICs to alleviate cost-specific barriers. Mobile and satellite clinics, as well as telehealth, are offered in these communities to provide access to services if geographical access is an issue [60]. Community health programs and health promoters have been used in both nations to enhance health literacy and improve access to care. Bilingual community navigators provide migrant patients with culturally tailored health education on income [66].

Future directions and recommendations 

The potential of telemedicine, artificial intelligence (AI), and mobile health solutions can bridge gaps in access to care, particularly in LMICs with limited healthcare infrastructure. Telemedicine can facilitate remote consultations and follow-ups, reducing the need for travel and enabling timely interventions [67,68], while at the same time reducing the costs associated with transportation, parking, and lodging to attend medical visits [69]. AI can assist in early detection through advanced image analysis and predictive modeling, potentially increasing diagnostic accuracy and efficiency [70,71]. Mobile health applications can promote awareness, remind patients of screening appointments [72], and facilitate data collection for ongoing research. Prospects for CRC include implementing personalized medicine, where treatments are tailored to the genetic profile of each patient, improving efficacy and reducing side effects [73]. Genetic screening can also identify high-risk individuals for targeted surveillance and preventive measures [71,74]. Lastly, novel therapies, such as immunotherapy and targeted molecular treatments, hold the potential to significantly improve survival rates and quality of life for CRC patients. Still, further research is needed to evaluate these treatments [75-77].

Governments and international organizations are essential in ensuring equitable access to CRC care. Policy recommendations must prioritize integrating cancer prevention into primary healthcare systems, ensuring early detection, reducing healthcare disparities, and ensuring cost-effective, comprehensive care that benefits the entire population [78]. Policies should focus on increasing funding for cancer research, prevention, and healthcare infrastructure to mitigate disparities between LMICs and HICs [79]. Furthermore, improving data collection and surveillance systems can help track disease prevalence and treatment outcomes, and inform policy decisions. Advocacy efforts should raise public awareness, reduce stigma, and promote healthy lifestyle choices to prevent cancer [80]. In addition, collaborative initiatives between the public and private sectors can foster innovation and resource sharing, while international organizations can provide technical support and funding to LMICs. By aligning policies with global health goals and ensuring equitable resource allocation, substantial progress can be made in reducing the burden of colon cancer worldwide. At the individual level, physicians need to be up to date on the diagnostic and treatment modalities and be aware of the psychosocial aspects of each patient and environment [81].

## Conclusions

This review highlights significant disparities in CRC screening and management between LMICs and HICs, with low-income regions facing limited access to screening and treatment. Tailored approaches, such as low-cost screening and community engagement, are important for improving outcomes in these areas. Global collaboration and innovative solutions like telemedicine, AI, and personalized medicine promise to bridge these gaps. To reduce the global burden of CRC, it is essential to integrate cancer prevention into primary healthcare, increase funding, and enhance data collection, ensuring equitable access to care and better health outcomes for all.
